# Galectin-1 Inhibitor OTX008 Induces Tumor Vessel Normalization and Tumor Growth Inhibition in Human Head and Neck Squamous Cell Carcinoma Models

**DOI:** 10.3390/ijms18122671

**Published:** 2017-12-09

**Authors:** Nathan A. Koonce, Robert J. Griffin, Ruud P. M. Dings

**Affiliations:** 1 Department of Radiation Oncology, University of Arkansas for Medical Sciences, Little Rock, AR 72205, USA; Nathan.Koonce@fda.hhs.gov; 2 National Center for Toxicological Research, Food and Drug Administration, Jefferson, AR 72079, USA

**Keywords:** galectin-1 inhibitor, OTX008, Avastin, Anginex, hypoxia, pimonidazole, vessel normalization

## Abstract

Galectin-1 is a hypoxia-regulated protein and a prognostic marker in head and neck squamous cell carcinomas (HNSCC). Here we assessed the ability of non-peptidic galectin-1 inhibitor OTX008 to improve tumor oxygenation levels via tumor vessel normalization as well as tumor growth inhibition in two human HNSCC tumor models, the human laryngeal squamous carcinoma SQ20B and the human epithelial type 2 HEp-2. Tumor-bearing mice were treated with OTX008, Anginex, or Avastin and oxygen levels were determined by fiber-optics and molecular marker pimonidazole binding. Immuno-fluorescence was used to determine vessel normalization status. Continued OTX008 treatment caused a transient reoxygenation in SQ20B tumors peaking on day 14, while a steady increase in tumor oxygenation was observed over 21 days in the HEp-2 model. A >50% decrease in immunohistochemical staining for tumor hypoxia verified the oxygenation data measured using a partial pressure of oxygen (pO_2_) probe. Additionally, OTX008 induced tumor vessel normalization as tumor pericyte coverage increased by approximately 40% without inducing any toxicity. Moreover, OTX008 inhibited tumor growth as effectively as Anginex and Avastin, except in the HEp-2 model where Avastin was found to suspend tumor growth. Galectin-1 inhibitor OTX008 transiently increased overall tumor oxygenation via vessel normalization to various degrees in both HNSCC models. These findings suggest that targeting galectin-1—e.g., by OTX008—may be an effective approach to treat cancer patients as stand-alone therapy or in combination with other standards of care.

## 1. Introduction

Agents that target the tumor microenvironment and inhibit angiogenesis have shown promise as therapeutics in solid tumors. Although anti–vascular endothelial growth factor (VEGF) agents like bevacizumab (Avastin^TM^, a humanized monoclonal antibody against VEGF; Genentech (South San Francisco, CA, USA)) are perhaps most discussed [1], many other compounds have been identified and are currently in various phases of clinical cancer trials [2,3]. Nevertheless, some angiogenesis inhibitors and vascular disruptors are ineffective or cause unwanted biological side effects [4], which underscores the need for more diverse compounds and/or treatment strategies. Although it is critical to recognize that anti-angiogenesis treatment can take the form of more than growth factor pathway inhibition alone, few other agents have made the clinical impact of Avastin.

Galectins are a family of carbohydrate-binding lectins that share a conserved carbohydrate recognition domain by binding to β-galactoside-containing glycoconjugates. Galectins promote cell-cell and cell-matrix interactions during cancer development and progression. Our recent results have validated (galectin-1) gal-1 as a viable cancer target with broad potential [5,6], as differential stromal elevation of gal-1 over the tumor parenchyma has been reported in several cancers, including those of head and neck, ovary, breast, brain, colon, skin, and prostate [7,8,9,10,11].

To date, most known galectin antagonists are glycomimetics and are derivatives or analogs of β-galactoside, targeting the canonical carbohydrate-binding site of galectins. These include aryl *O*- and *S*-galactosides and lactosides [12,13], carbohydrate-based triazoles and isoxazoles [14], *O*-galactosyl aldoximes [15], phenyl thio-β-d-galactopyranoside analogs [16], thioureido *N*-acetyllactosamine derivatives [17], talosides [18], and various multivalent sugar-based compounds [19]. We have previously reported on the design of Anginex, a 33mer amphipathic peptide that targets gal-1 [5,20]. While antibodies and peptides can be effective, small molecules tend to be advantageous in regards to bioavailability, immunogenic activation, degradation, and ability to scale up. We employed the calix[4]arene scaffold for the development of a peptidomimetic of Anginex because it approximated the molecular dimensions and surface topology of key spatially-related amino acid residues in Anginex [21]. We identified OTX008 to have more promising biological properties than Anginex [22] and discovered that OTX008 is an allosteric inhibitor of gal-1 as it binds at a different site than the canonical carbohydrate binding site [23].

Previous studies have identified gal-1 as a negative prognosticator in oral squamous cell carcinoma (OSCC) and human head and neck squamous cell carcinoma (HNSSC) [24,25]. Here, we investigated whether OTX008 can normalize tumor vasculature resulting in improved tumor oxygenation and subsequent tumor growth inhibition as compared to Anginex and Avastin in two HNSSC models, namely SQ20B and HEp-2. Whereas SQ20B has mutated *p53* and is resistant to radiation but sensitive to OTX008, HEp-2 is an HNSSC epithelial cell line derived from human epidermoid carcinoma of the larynx, which is resistant to both standard radiochemotherapy regiments as well as OTX008 [26].

## 2. Results and Discussion

### 2.1. OTX008 Increases Overall Tumor Oxygenation

**Figure 1:**
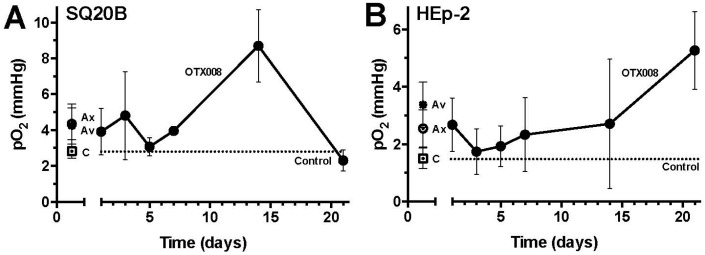
The effect of OTX008 on global tumor pO_2_ over time. Global tumor pO_2_ is transiently increased by OTX008 in SQ20B (**A**) and HEP2 (**B**) tumors as measured by fiber-optic oxygen sensor. Treatments: Control (C; -□-), OTX008 (-●-), Anginex (Ax; -○-), and Avastin (Av; -✱-). Points, average mean (± SEM) pO_2_ value derived from the stabilized reading over a 60 s period at one location (four to seven mice per day per experimental group). The dotted line represents the average pO_2_ mean of the control.

Repeated daily treatment with OTX008 resulted in an increased overall tumor oxygenation (pO_2_) up to day 14 of treatment (compared with vehicle-treated mice) in the SQ20B tumor model (Figure 1A). After day 14, tumor oxygenation decreased to levels in line with those measured in vehicle-treated mice. In contrast, in the HEp-2 tumor mouse model overall tumor oxygenation levels were steadily elevated throughout OTX008 treatment as compared to vehicle (control) treated mice (Figure 1B). In both models, this improvement in tumor oxygenation was comparable with the average increases induced by either Anginex or Avastin (Figure 1). Individual tumor oxygenation values for Anginex and Avastin in time for either model can be found in Figures S1 and S2. Overall, the results in the SQ20B and the HEp-2 models reiterated the theory that transient or sustained vessel normalization is not solely a VEGF-dependent phenomenon and moreover is seen in multiple solid tumor models including HNSSC. However, it is also interesting to note that the “tumor oxygenation window” did not occur in all tumor models, as the HEp-2 model displayed improved oxygenation throughout the study. Previously, other tumor mouse models and human clinical data have shown that this window occurs earlier, around day 3 through 5 after commencement of treatment [11,27]. The tumors in these previous studies were in general larger at the beginning of the treatment, which may have accounted for the more rapid change in oxygenation levels. The measurement of tumor oxygenation with various semi-invasive approaches may also influence the results obtained. Specifically, in the current study we sampled partial oxygenation pressure with an inserted fiber optic probe over several weeks at 1 to 3 points in each tumor were viable baseline oxygenation levels were found, thus intentionally avoiding necrotic or anoxic regions. Other invasive probes collect 50–100 point measurements and thus may be more influenced by the amount of necrotic volume being sampled [11,27]. It is also likely that this window varies due to origin, location, or growth rate of the particular cancer.

### 2.2. OTX008 Inhibits Tumor Growth without Apparent Toxicity

**Figure 2:**
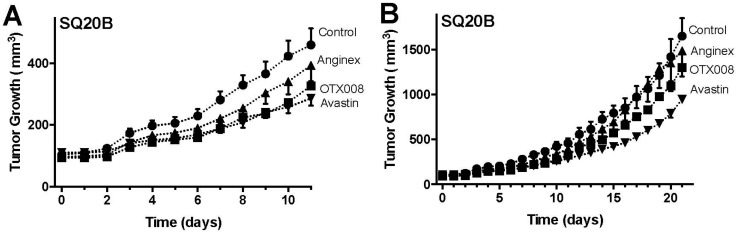
OTX008 inhibits SQ20B tumor growth in mice. (**A**) SQ20B tumor growth during the first 11 days and treatment with Control (vehicle), Anginex, OTX008 and Avastin; (**B**) SQ20B tumor growth during the whole treatment span with Control (vehicle), Anginex, OTX008 and Avastin. Treatments: Control (vehicle; -●-), Anginex (i.p. 10 mg/kg, q1d×21; *n* = 7; -▲-), OTX008 (i.p. 10 mg/kg, q1d×21; *n* = 7; -■-), and Avastin (i.v. 10 mg/kg, q14d×2; *n* = 7; -▼-). Data points represent means ± SEM (*n* = 7 each group).

**Figure 3:**
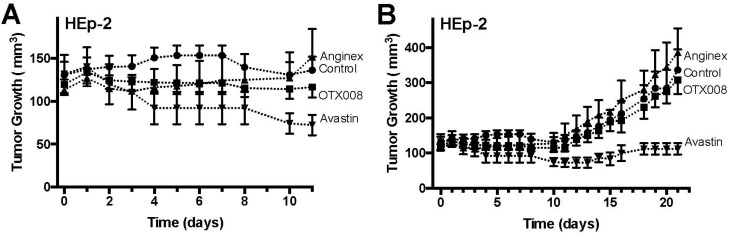
OTX008 inhibits HEp-2 tumor growth in mice. (**A**) HEp-2 tumor growth during the first 11 days and treatment with Control (vehicle), Anginex, OTX008 and Avastin; (**B**) HEp-2 tumor growth during the whole treatment span with Control (vehicle), Anginex, OTX008 and Avastin. Treatments: Control (vehicle; -●-), Anginex (i.p. 10 mg/kg, q1d×21; *n* = 7; -▲-), OTX008 (i.p. 10 mg/kg, q1d×21; *n* = 7; -■-), and Avastin (i.v. 10 mg/kg, q14d×2; *n* = 7; -▼-). Data points represent means ± SEM (*n* = 7 each group).

In a subsequent set of experiments, mice inoculated with SQ20B or HEp-2 tumor cells were randomized and treated with OTX008 (qd, 10 mg/kg, i.p. *n* = 7), Anginex (qd, 10 mg/kg, i.p. *n* = 7) or Avastin (once every 14 days, 10 mg/kg, i.v. *n* = 7) starting when tumors reached the size of ~100 mm^3^. In the SQ20B model OTX008 reduced tumor growth on average 25%, up to 35% on certain days (Figure 2). This inhibition was comparable to the effects of Avastin in this model and slightly better than Anginex (Figure 2). In the HEp-2 tumor model this was less pronounced, whereas Avastin caused tumor growth stabilization (Figure 3). As shown by Astorgues-Xerri et al., HEp-2 is inherently resistant to radiochemotherapy including OTX008 [26], which could explain the relatively limited HEp-2 tumor growth inhibition by OTX008. Additionally, in the current study OTX008 and Anginex was administered i.p. daily. As compared to previous reports, the data suggests that administering OTX008 or Anginex bis in die (BID) i.p. or subcutaneous (s.q.) continuously by osmotic mini-pump will result in greater tumor growth inhibition [20,22,28,29].

**Figure 4:**
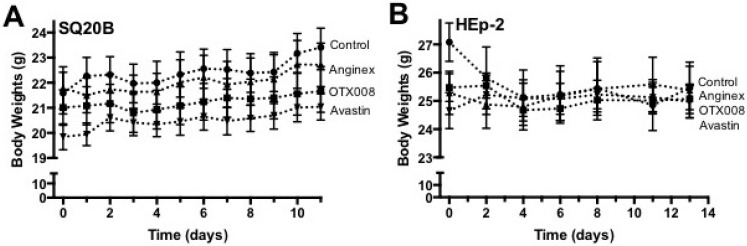
OTX008 does not show any sign of apparent toxicity. (**A**) In the SQ20B OTX008, Anginex and Avastin do not show any signs of apparent toxicity as measured by body weight fluctuation during treatment; (**B**) In the HEp-2 OTX008, Anginex and Avastin do not show any signs of apparent toxicity as measured by body weight fluctuation during treatment. Treatments: Control (vehicle; -●-), Anginex (i.p. 10 mg/kg, q1d×21; *n* = 7; -▲-), OTX008 (i.p. 10 mg/kg, q1d×21; *n* = 7; -■-), and Avastin (i.v. 10 mg/kg, q14d×2; *n* = 7; -▼-). Data points represent means ± SEM (*n* = 7 each group).

In both tumor models studied, prolonged treatment with OTX008, Anginex, or Avastin revealed no signs of toxicity as assessed by unaltered behavior and normal weight gain during experiments (Figure 4). Upon autopsy, macro- and microscopic morphologies of internal organs were also observed to be normal within all experimental groups of animals.

### 2.3. OTX008 Reduces Tumor Hypoxia and Improves Pericyte Coverage

**Figure 5:**
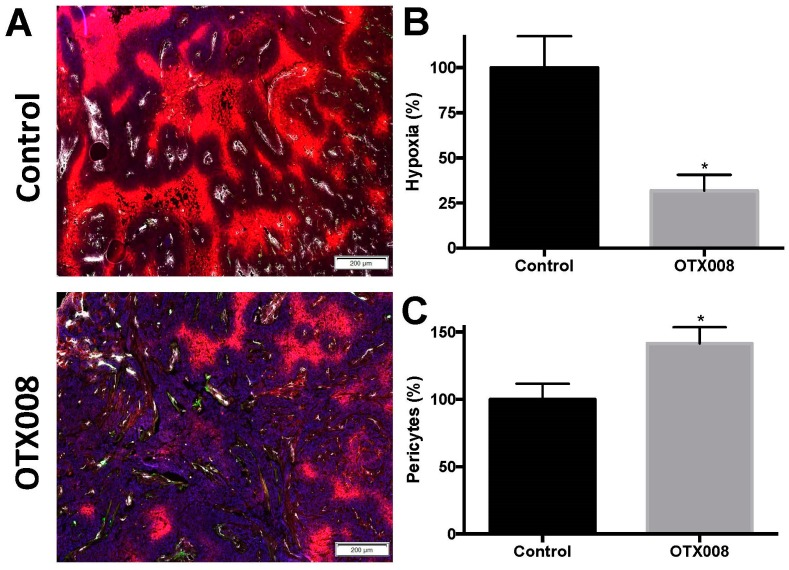
Hypoxia and pericyte content in SQ20B tumor tissue after OTX008 treatment. (**A**) Immunofluorescence analysis of SQ20B tumors cross-sections from OTX008 or vehicle treated animals at 14 days after the beginning of OTX008 therapy. Microvessel density is revealed by CD31 antibody staining (white), pericytes by alpha-smooth muscle actin (αSMA; green) and hypoxia by pimonidazole (red). Magnifications as indicated; (**B**) Quantification of hypoxia by pimonidazole positive regions; (**C**) Quantification of pericytes by α -SMA positive cells. Data is depicted as means ± SEM and normalized to the controls, set at 100%; * *p* < 0.05 (Student’s *t*-test).

OTX008 treatment resulted in a decrease of SQ20B tumor hypoxia, as measured by pimonidazole. Pimonidazole, a substituted 2-nitroimidazole, is preferentially reduced in hypoxic viable cells and forms irreversible protein adducts, which have been optimized for detection with immunohistochemistry. On average, OTX008 reduced hypoxia by ~70% in SQ20B tumors (Figure 5). These immunofluorescence results are in line with the improved oxygenation levels as determined by direct measurement of the partial pressure of oxygen (Figure 1). Besides the improvement of tumor oxygenation, vessel normalization was correlated with an improvement of pericyte coverage. As shown and quantified in Figure 5, OTX008 increased the amount of pericyte-covered vessels by ~40% in SQ20B tumors.

Overall, our results show that OTX008 can transiently increase overall tumor oxygenation via vessel normalization with varying kinetics and magnitude/duration of effects as tumors progress. Since Avastin, in combination with chemotherapy is already an Food and Drug Administration approved protocol for treatment of several tumor types [30], a gal-1 therapeutic such as Anginex or OTX008 could possibly avoid the noted systemic effects that Avastin usage promotes [2,3,4]. Moreover, gal-1 inhibitors may achieve a more permissive tumor microenvironment status for improved radiation or chemotherapeutic treatment.

Additionally, non-peptidic topomimetic OTX008 might be advantageous over a biologic such as Avastin based on the drug properties. Whereas biologics, antibodies included, are relatively large proteins (up to 150 kDa) they differ from small molecules in their cost, production, administration, and clinical efficacy. Larger proteins and antibodies are predominantly more difficult to produce and characterize as protein folding and tertiary structure are essential for their efficacy, resulting in increased costs. Due to these inherent challenges, specificity, scale up, purity, and batch consistency can also be more demanding [31]. Moreover biologics, Avastin included, are generally administered intravenously as they are likely to be digested when taken orally [32]. Small molecules or synthetics chemicals on the other hand are often orally available and absorbed into the bloodstream via the intestinal wall due to their small size and chemical composition. Additionally, larger proteins are assumed more likely to be immunogenic and less efficient in tissue penetration, tumor retention, and blood clearance than small molecules [32]. 

Subsequently, it would be pertinent to elucidate whether the increase in oxygenation caused by OTX008 enhances the effect of radiation therapy in vivo, especially if radiation at a large dose per fraction could be applied at the peak of tumor oxygenation observed. Furthermore, there may be opportunities to enhance the effects of various chemotherapeutic agents due to the improved physiological state of tumors during OTX008 treatment. By administering chemotherapy during the OTX008 oxygenation window, we would expect to maximize drug penetration/diffusion and overall coverage of the tumor. Recent preclinical and clinical reports on gal-1 playing a principal role in tumor progression, therapeutic resistance, and metastatic potential of multiple tumor types [33,34,35] highlight the translational potential of OTX008 and other gal-1 inhibitors in development.

## 3. Materials and Methods

### 3.1. Test Article and Control Reagents

OTX008 (also known as PTX008 or 0118) was reconstituted in H_2_O at a stock concentration of 1 mg/ml and stored at −80 °C [22]. Anginex was manufactured and obtained from BioMedical Genomics Center at the University of Minnesota (Minneapolis, MN, USA) and formulated identical to OTX008 and stored at 2–8 °C, as before [36]. OTX008 and Anginex were given daily for 21 days (q1d×21) in alternating intraperitoneal (i.p.) locations at 10 mg/kg.

Avastin™ (bevacizumab, a humanized monoclonal antibody against VEGF) was obtained from the University of Arkansas for Medical Sciences (UAMS) pharmacy, manufactured by Genentech). The antibody is provided at a stock concentration of 25 mg/mL and stored at 2–8 °C. Avastin was given once every 14 days (q14d×2) intravenously (i.v.) at 10 mg/kg.

### 3.2. Tumor Models

Experiments were approved by the University of Arkansas for Medical Sciences Institutional Animal Care and Use Committee (Animal Usage Protocol #3157; 05 17 2013) and performed in accordance with relevant regulations and guidelines. For the xenograft models, female athymic nude mice (*Nu/Nu*, 5–6 weeks old) were inoculated into the right flank s.c. with tumor cells and when tumors reached a size of ~100 mm^3^, animals were randomly assigned to treatment groups and administration of study drug was commenced. In total, 92 animals were inoculated and a total of 76 tumor-bearing animals were selected for randomization before the initiation of the intervention.

SQ20B—radio-resistant HNSCC SQ20B carries a *p53* mutation and has a constitutively active mutation in epidermal growth factor receptor (EGFR) resulting in robust Akt (protein kinase B) signaling [26,37,38,39]. It was shown previously that this cell line is sensitive to OTX008 in different in vitro and in vivo experiments [40].

HEp-2—an epithelial cell line derived from human epidermoid carcinoma of the larynx. This cell line is more resistant to current standard chemotherapeutic agents and it is more resistant in vitro to OTX008 as compared to HNSCC SQ20B [40].

### 3.3. Tumor Growth

Subcutaneous tumors were measured with a caliper prior to starting treatment (prior to randomization into treatment groups) and then once a day during treatment. Tumor volumes were calculated based on the equation (a^2^ × b)/2 where a is the shortest and b is the longest diameter of the tumor (width or length) [39].

### 3.4. Tumor Oxygenation Studies

Tumor partial pressure of oxygen (pO_2_) was measured using a fiber-optic oxygen sensor (Oxylite™, Oxford Optronix, Oxford, UK) [41]. The probe was inserted via a needle track to approximately the center of the three dimensional tumor mass and readings were recorded for 3 min. Final values were based on the stabilized value over a 60 s period at one location. Once tumors grew to a larger size, the probe was moved to one or two more locations and readings recorded over 3 min at each position. The readings were based on four to seven mice per day per experimental group.

### 3.5. Tumor Vessel Density and Pericyte Staining

Extracted tumors were either fixed in 10% formalin or embedded in tissue freezing medium and snap frozen in liquid nitrogen. Formalin-fixed tumors embedded in paraffin or frozen tumors were cut into 5 µm sections. After rehydration and antigen retrieval, the slides were stained for either vessel density (CD31 from BD Pharmingen (San Jose, CA, USA); 2nd Ab αRat-647) or pericytes (anti-alpha-smooth muscle actin, αSMA-FITC from Sigma-Aldrich (St. Louis, MO, USA)) [22].

### 3.6. Tumor Hypoxia

An i.p. injection of 60 mg/kg pimonidazole was given to each mouse prior to euthanasia. Pimonidazole, a substituted 2-nitroimidazole, is preferentially reduced in hypoxic viable cells and forms irreversible protein adducts, which have been optimized for detection with immunohistochemistry [42]. At 1 h post-injection, the mice were euthanized, then the tumor dissected and immediately either fixed in 10% formalin or snap frozen. Following sectioning of tumor tissue, a FITC-conjugated monoclonal antibody against protein adducts of pimonidazole was added (hypoxyprobe-1 MAb; Chemicon International, Burlington, MA, USA); 2nd Ab αRabbit-594). Images of the section were acquired and subsequently digitally and differentially quantified by morphometric analysis [11].

### 3.7. Body Weight

As an indirect measure of general toxicity, body weights were measured prior to starting treatment and once daily during treatment. At the end of the study by autopsy, macro- and microscopic morphologies of internal organs were inspected and recorded in all experimental groups of animals [22].

## Supplementary Materials

Supplementary materials can be found at www.mdpi.com/1422-0067/18/12/2671/s1.
