# Supplementary material for: Galectin-1 Inhibitor OTX008 Induces Tumor Vessel Normalization and Tumor Growth Inhibition in Human Head and Neck Squamous Cell Carcinoma Models
Source: Int J Mol Sci. 2017 Dec 9;18(12):2671. doi: 10.3390/ijms18122671 (PMC5751273; doi:10.3390/ijms18122671)
Supplement: Supplementary file 1 [file ijms-18-02671-s001.pdf]

Supplemental Information

Communication

Galectin-1 Inhibitor OTX008 Induces Tumor Vessel Normalization and Tumor Growth Inhibition in Human Head and Neck Squamous Cell Carcinoma Models

Nathan A. Koonce [1,2], Robert J. Griffin [1,*], and Ruud P. M. Dings [1,*]

[1] Department of Radiation Oncology, University of Arkansas for Medical Sciences, Little Rock, 72205 AR, USA

[2] National Center for Toxicological Research, Food and Drug Administration, Jefferson, 72079 AR, USA; Nathan.Koonce@fda.hhs.gov

* Correspondence: rjgriffin@uams.edu (R.J.G.); rpmdings@uams.edu (R.P.M.D.); Tel.: +1-501-526-7873 (R.J.G.); +1-501-526-7876 (R.P.M.D.); Fax: +1-501-526-5934 (R.J.G.); +1-501-526-5934 (R.P.M.D.)

Received: 6 November 2017; Accepted: 5 December 2017; Published: 9 December 2017

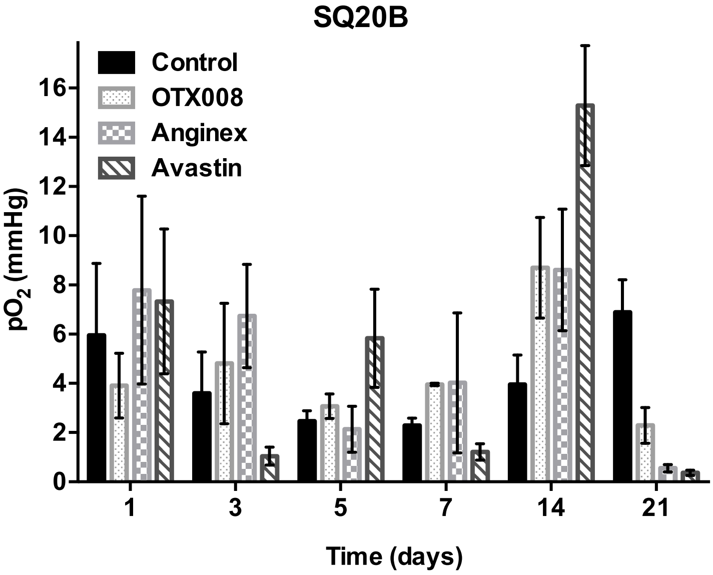

Figure S1. The effect of OTX008, Anginex and Avastin on global SQ20B tumor pO_2 over time. Points, average mean (± SEM) pO_2 value derived from the stabilized reading over a 60 seconds period at one location (4–7 mice per day per experimental group).

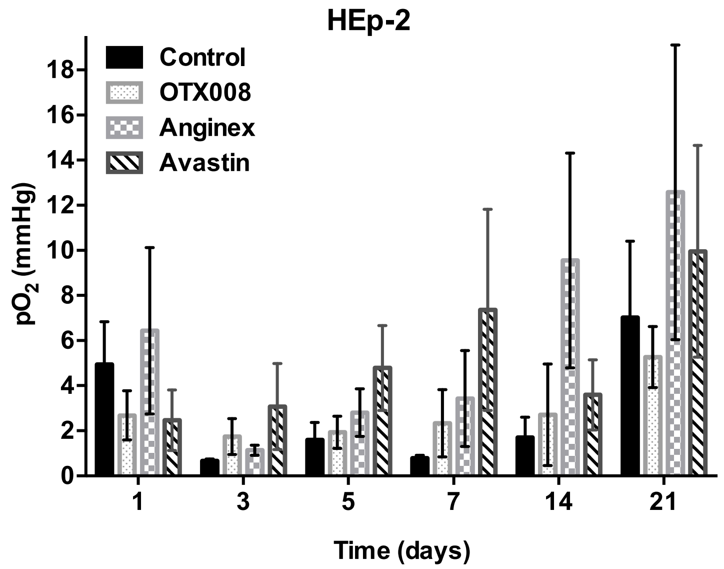

Figure S2. The effect of OTX008, Anginex and Avastin on global HEP-2 tumor pO_2 over time**.** Points, average mean (± SEM) pO_2 value derived from the stabilized reading over a 60 seconds period at one location (4–7 mice per day per experimental group).
